# Effect of Seasonal Decrease in Temperature on the Content and Composition of Guayulins in Stems of Guayule (*Parthenium argentatum*, Gray)

**DOI:** 10.3390/plants10030537

**Published:** 2021-03-12

**Authors:** Juana Rozalén, M. Mercedes García-Martínez, Maria Engracia Carrión, Amaya Zalacain, Horacio López-Córcoles, Manuel Carmona

**Affiliations:** 1 Catedra de Química Agrícola, Escuela Técnica Superior de Ingenieros Agrónomos y de Montes, Universidad de Castilla-La Mancha, Campus Universitario s/n, 02071 Albacete, Spain; juana.rozalen@uclm.es (J.R.); Amaya.Zalacain@uclm.es (A.Z.); 2 Food Quality Research Group, Institute for Regional Development (IDR), Universidad de Castilla-La Mancha, 02071 Albacete, Spain; mariamercedes.garcia@uclm.es; 3 Food Technology Lab, School of Architecture, Engineering and Design, Universidad Europea de Madrid, C/Tajo s/n, 28670 Villaviciosa de Odón (Madrid), Spain; mengracia@linkidi.com; 4 Instituto Técnico Agronómico Provincial de Albacete, ITAP, Parque Empresarial Campollano, 2ª Avenida, 61, 02007 Albacete, Spain; hlc.itap@dipualba.es

**Keywords:** guayule, resins, guayulins, sesquiterpenes, stem, leaf

## Abstract

The guayulins are a family of sesquiterpene compounds that consist of an isoprenoid nucleus substituted either by *trans*-cinnamic or *p*-anisic acid, and are present only in the resinous fraction of the rubber plant guayule (*Parthenium argentatum*, Gray). While the natural role of the guayulins remains enigmatic, they may serve as a defense function against other plants or herbivores by virtue of the accumulation of cinnamic acid. Prior research has suggested seasonal variation in guayulin content, which has been shown to decrease as winter arrives in two different varieties. In the present study, the effect of guayulins has been evaluated in 13 different accessions cultivated under the same conditions during autumn. A general reduction in guayulin content was found in the stems from all varieties between the September and November harvest, which was accompanied by an increase in the resin content. With respect to individual guayulins, while guayulin A was the most prominent member during most of the year, guayulin C had more prominence when temperature started to decrease. In this seasonal period, the production of each member of the guayulin family in the leaves was very balanced.

## 1. Introduction

**Figure 1:**
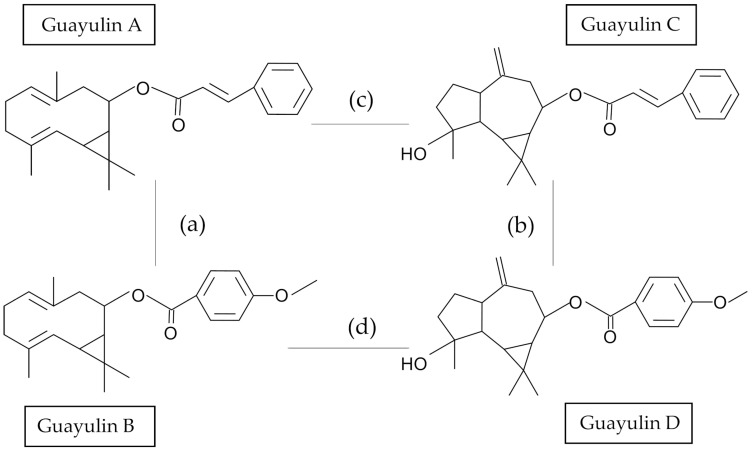
Structure of the four main guayulins, and the relationship between them: (**a**) guayulins related with a bicyclogermacrene structure (Guayulins A and B); (**b**) guayulins related with an aromadendrane structure (Guayulins C and D); (**c**) guayulins esterified by *trans*-cinnamic acid (Guayulins A and C); (**d**) guayulins esterified by *p*-anisic acid (Guayulins B and D).

The guayulin family of sesquiterpene secondary metabolites comprises four known members, guayulin A–D [1,2], although several other members may exist [3,4]. Guayulin A was first isolated in 1911 and its structure was determined some years later [5,6]. Guayulins consist of an isoprenoid nucleus, which may be partheniol, with a bicyclogermacrene structure (Guayulins A and B), or its derivative compound with an aromadendrene structure (Guayulins C and D) [7], esterified either by *trans*-cinnamic acid (Guayulins A and C), or *p*-anisic acid (Guayulins B and D) [3,4] (Figure 1). Guayulins have been described in high concentration in the resin component of the rubber plant commonly known as guayule (*Parthenium argentatum*, Gray), a perennial shrub native to the Chihuauhan desert region of northern Mexico and Texas that does not produce the pseudoguaianolide sesquiterpene lactones found in almost all other species of the genus Parthenium [8].

Sesquiterpenes lactones have attracted much scientific attention because of their functional diversity and abundance, and they are important taxonomic markers in the family Asteraceae to which the genus Parthenium belongs [8,9]. Sesquiterpenes lactones display antimicrobial, antifeedant, growth-modifying and allelopathic activities [10,11], a virtue of to their high reactivity. This is likely related to the α-methylene moiety [8,10], a functional group that the guayulins lack. Looking to the other guayule-derived extracts i.e., latex or rubber, there is possibly a major reason for its commercial exploitation as it is known to be hypoallergenic due to the lack of the protein antigens present in *Hevea brasiliensis* natural rubber latex [12].

In contrast to sesquiterpenes lactones, much less is known about the biology and significance of the guayulins. Some authors have proposed that they could be a defense system of guayule against other plants due to the accumulation of cinnamic acid [9], or against herbivores [13] or insects [5]. Based on the hypotheses of Prokofiev [14] and Bonner and Galston [15], who suggested that there is a single common precursor in guayule that gives rise to terpenes or natural rubber depending on the environmental conditions, Mears and Larson [9] questioned whether the chemical traits of guayule could be causal. Indeed, it is the only species of the genus *Parthenium* that produces rubber but not sesquiterpenes lactones [8].

In a previous study developing analytical techniques to determine guayulin content [4], it was observed that guayulin A content significantly decreased during autumn in two guayule accessions with a high genotypic diversity (triploid 11619 and hybrid CAL-1) [16], but conversely, the resin content was higher at this stage [4]. Such guayulin A decrement that has been reported to be seasonally variable [17], coincided with the activation of natural rubber production [18,19,20]. The finding shown by these two accessions [4] has allowed one to identify a pattern of behavior for previous studies [17], although only with two accessions, which is not enough to demonstrate such behavior.

Besides, while the stem content of guayulin A was lower than that of other guayulins, particularly guayulin C, the opposite was seen in the leaf, where the content of guayulin A was high and of the same order as that of guayulin C [4]. This is interesting, because the precursor of natural rubber is believed to be formed in the leaf, and is then assembled in the epithelial cells of the resin channels in the stem and roots [21], and because prior studies suggested that defoliation due to frost damage is responsible for the reduction in rubber yield during winter [20,22]. Guayulins C and D, which are believed to be formed from guayulins A and B [23], have also been considered as potential commercial products [4], as their concentration in the resin increases over time.

The purpose of the present study was to establish: (1) whether the decline in the content of one or more guayulins in the stems during the autumn is a general behavior in multiple guayule accessions grown under the same conditions; (2) whether the drop in guayulin content has any impact on the resin yield; (3) whether there is any relationship between the potential drop in the guayulin content and the genetic origin of the various accessions; and finally (4), to question what occurs in the leaves and relate it to what happens in the stems.

## 2. Results

**Figure 2:**
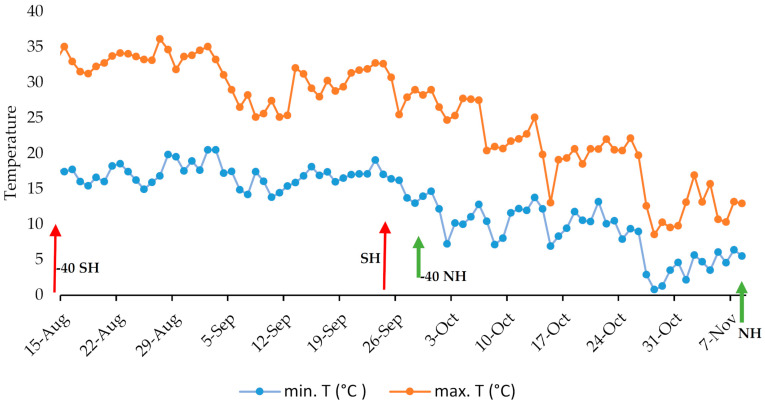
Maximum and minimum temperatures between 40 days before harvest date (−40SH red, −40NH green) and the harvesting dates on September 25th (SH red) and November 8th (NH green) of 2018.

A significant reduction in guayulin content occurs at the beginning of winter in two distinct guayule accessions [4]. It is well known that the accumulation of natural rubber in guayule increases significantly in the fall or early winter, when the temperature drops [19,20]. In this case, a more extensive study of this phenomenon was performed using a greater number of accessions with the aim of establishing representative behavior models monitoring the weather conditions to determine their effect on guayule behavior (Figure 2).

According to the seven-day mobile media, the minimum temperature (minT_07, Figure 2) from 17 August, 40 days before the 25 September harvest (−40 SH) date (Figure 2, red arrow), to 2 October was steady at 16 °C. The temperature then dropped in the first few days of October, coinciding with the beginning of the second 40-day period before the 8 November harvest date (−40 NH, Figure 2, green arrow). This drop led to a similar plateau at ~10 °C, and the temperature again dropped just ten days before the harvest to bring the night temperatures down to ~5 °C, specifically to 6.4 °C on the night of the 7 November, just prior to the harvesting day. These night-time temperatures (7–10 °C) are similar to those reported by some authors as necessary to activate rubber synthesis [24,25]. Later studies that considered plant age for cold induction suggested that higher minimum temperatures also trigger rubber accumulation [19,26].

### 2.1. Characterization of Total Resin and Total Guayulin Content in Stems

**Figure 3:**
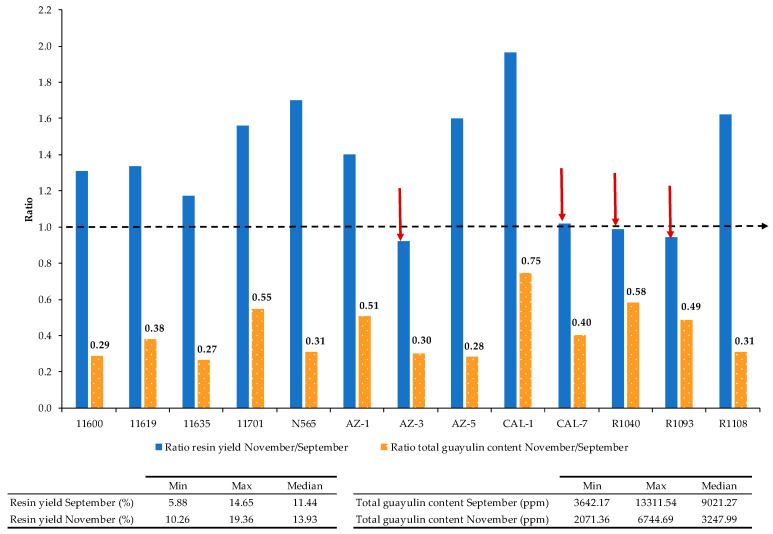
Relation of resin yield and total guayulins content in guayule stems between November and September in different guayule accessions. Bars represent the ratio of resin percentage (blue color) to guayulins content (orange color) in November versus September. The red line represents the same proportion in both harvesting times, whereas the red arrows highlight the guayule accessions without a significant decrease in resin content in the November harvest. Orange arrow represents the guayule accession in which total guayulins content does not decrease in the November harvest.

There is extensive knowledge about the temperature conditions necessary to actively promote the synthesis of natural rubber in guayule [19,24,25], but little is known about the resin content and much less the compounds that it contains. A loss of resin content from November to January has been previously addressed [20], and the guayulin content could decrease with the arrival of winter [4]. Analysis of the 13 accessions revealed that the yield of resin was higher in the November harvest than in the September harvest, with a minimum of 10.3% and maximum of 19.4% (median of 13.9%) (Figure 3). The opposite was seen for the total guayulin content, with the minimum and maximum values measured in November one-third of those in September, showing a median value of ~3 g/kg dry weight and reaching ~9 g/kg in September (Figure 3).

Closer inspection of the resin yield ratio November/September by accession revealed a general behavior with some exceptions (Figure 3), and highlighted a tendency for increased resin content in the period of September–November, as reported by other authors [27]. The resin content increase was significant in nine of the 13 accessions. By contrast, the resin content in CAL-7 increased only slightly, and three accessions showed less resin content in the November harvest (AZ-3, R1040, R1093), although not significant. In these four cases, the decrease in total guayulin content was much higher than the decrease in resin content (guayulin content varied between 30% and 58% of that in September). Overall, these results show that the decrease in the guayulin content is not due to a decrease in the quantity of resin.

**Figure 4:**
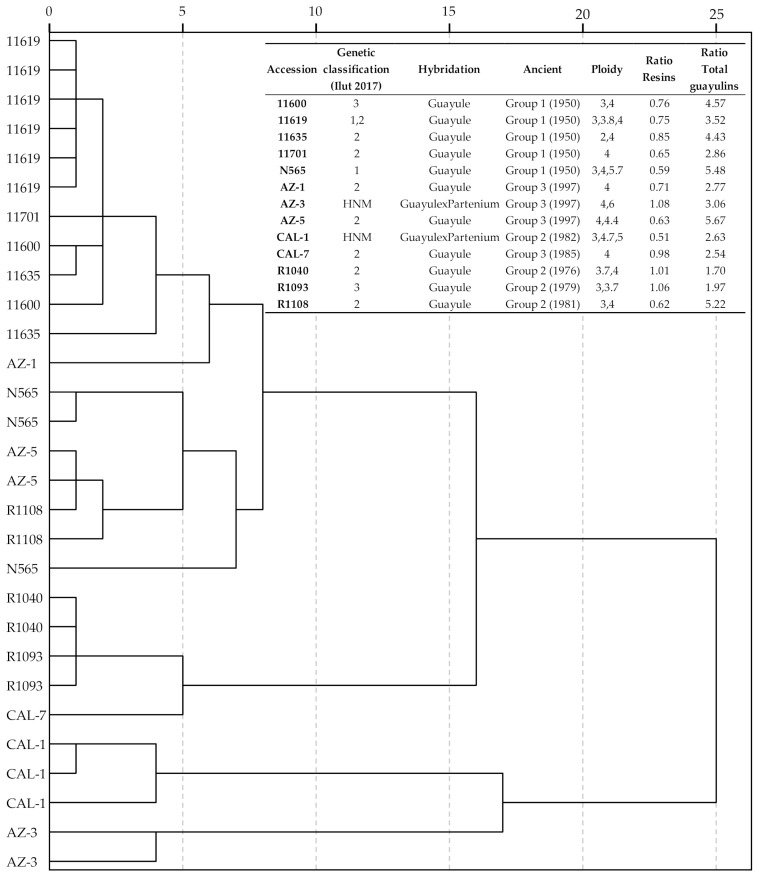
Relationship of resin and total guayulins content with accession ploidy and genetic origin according to Ilut et al. [16].

A comparative analysis of the values obtained for total resin and guayulin content based on the genetic origin and ploidy for these accessions established by Ilut et al. [16] is shown in Figure 4.

In this figure, some accessions are repeatedly represented, as all of the combinations among the genetic classification according to Ilut et al. [16] and the potential ploidy have been considered. The following ancient groups have been established according to Ilut et al. [28]: 1942–1960, 1961–1982 and 1983–2008. The most remarkable aspect of this analysis was that hybrids AZ-3 and CAL-1, which belong to the same ancient group but come from different Parthenium species [16], were clearly separated from the remaining accessions. These two hybrids did not show significant differences in resin and total guayulin ratios (Figure 3). A second group comprises CAL-7, R1093 and R1040, which did not show significant differences in resin content between September and November (Figure 3). They are pure guayule accessions with similar resin and total guayulin ratios, and the phylogenetic analysis performed by Ilut et al. [16] demonstrates that tetraploid samples from CAL-7 and R1040 are very closely related. The remaining accessions group together.

### 2.2. Characterization of Guayulin Profile in Stems

**Figure 5:**
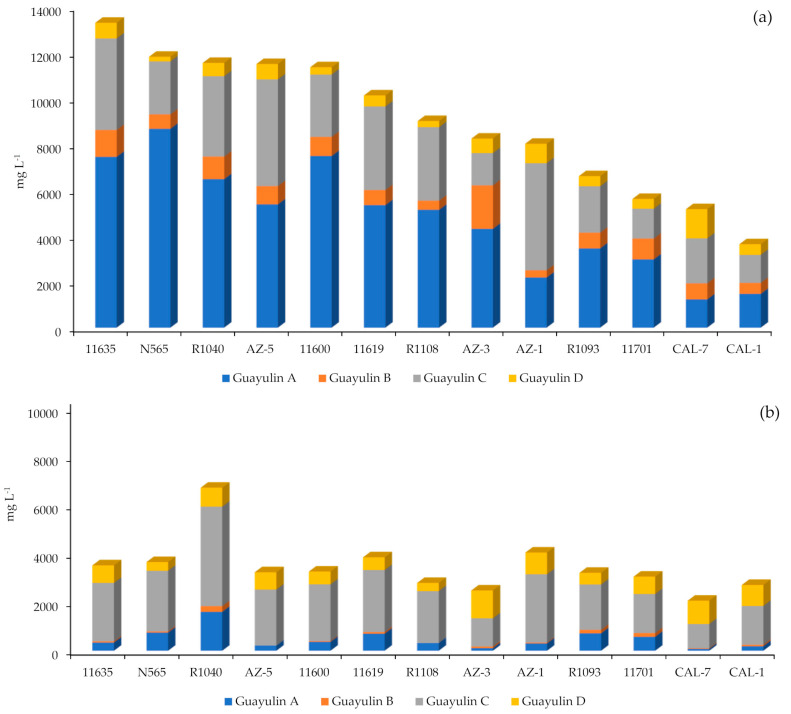
Quantitation of guayulins in the September and November harvest (expressed as mg L^−1^ of guayulin): (**a**) in September; (**b**) in November.

When the detailed behavior of every guayulin was analyzed (Figure 5), six of the 13 studied accessions showed a higher production of guayulins in the September sampling than in November (Figure 5a).

Five of these (11600, N565, 11635, 11619 and 11701) are historical guayule accessions derived from the Emergency Rubber Project (ERP, 1942–1946) with an origin in the ancestral 4265-I accession [16]. In addition to the aforementioned evident lower amount of guayulin content in the month of November, a clear change in the proportion of individual guayulins was observed (Figure 5a,b). Guayulin A was the major guayulin in in all accessions when collected in September, except for AZ-1 and the CAL-7 hybrid, and it was taken over in November by guayulin C (Figure 5a,b). Several authors have postulated that guayulin C is formed by oxidation of guayulin A, both sharing the cinnamic acid (Figure 1) [17,23]. Therefore, a relationship would be expected between both guayulins, as was proved for this pair of compounds and for the guayulin B/D pair (Table 1). These relationships varied according to the accession, but with overlapping and common ranges: ratio A/C (min = 0.47; max = 3.74; median = 1.71) and ratio B/D (min = 0.37; max = 3.22; median = 1.57).

Less expected would be a relationship between compounds that have the same core but different moieties (Figure 1, cinnamic acid or *p*-anisic acid); that is, between guayulins A and B and guayulins C and D (Table 1). Nevertheless, in this case the values depended much more on the type of accession because although both relationships overlapped, they did so in a much greater range (min = 1.54; max = 13.76). These values could be integrated into those described by Teetor et al. [3]. These authors found that the ratio of guayulin A to B is much lower in the newly developed varieties AZ-1 and AZ-3, specifically, between 3.7 and 5.2 times depending on the thickness of the stem (in our case 2.3 and 6.9 times for the same accessions), than in the older variety 11591 (20:1 to 40:1). A decline in guayulin content was common in the November sampling. R1040 and R1093 accessions, corresponding to wild varieties harvested in Mexico (Coahuila and Durango) in the 1970s [16], and the AZ-1 line developed in the 1980s and 1990s derived from 4265-X [29] and 4265-XF (a selection of 4265-X), were similar to the five aforementioned Emergency Rubber Project (ERP) accessions as the largest producers of guayulins at that phenological stage. In addition to the general decline in guayulin content and the greater relative content of guayulin C, a disconnection in the relationships that existed in the month of September was observed (Table 1). On the one hand, the relations of isomerization (guayulin A to C, and guayulin B to D), decreased considerably to a narrow range (min = 0.03; max = 0.39) due to the great reduction of the content of guayulins A and B; to the extent that guayulin B became undetectable in the accessions AZ-5 and R1108. On the other hand, the relationships guayulin A/B and guayulin C/D no longer overlapped (Table 1). Although the content of guayulins A and B had dropped considerably, they still appeared to be related to one and other, following the same range of variation between accessions as in September, suggesting that as one decreases, the other does so in the same proportion. By contrast, the guayulin C/D ratio oscillated in a lower and narrower range of values (min = 1.02; max = 6.99) than during the month of September (Table 1). To evaluate whether this change in behavior was due to the decrease in one of the guayulins or the increase in the other, the content of each guayulin at the two harvesting times was compared (Table 2).

The results shown in Table 2 reveal that the profile change in the guayulin C/D ratio (Table 1) was principally due to the increase in guayulin D in November with respect to September, with the exception of the CAL-7. Conversely, guayulin C content decreased in November compared with September, except in accessions N565, R1040, 11701 and CAL-1. In the case of these four accessions, the increase in guayulin D was even greater, which made the ratio lower (Table 2). While the median for the guayulin C content for the November/September ratio was close to 0.8, that of guayulin D exceeded the value of 1.2 (Table 2) for all accessions. The decrease for guayulins A and B was very pronounced (median 0.09 and 0.08, respectively).

### 2.3. Characterization of Guayulin Profile in Leaves

**Figure 6:**
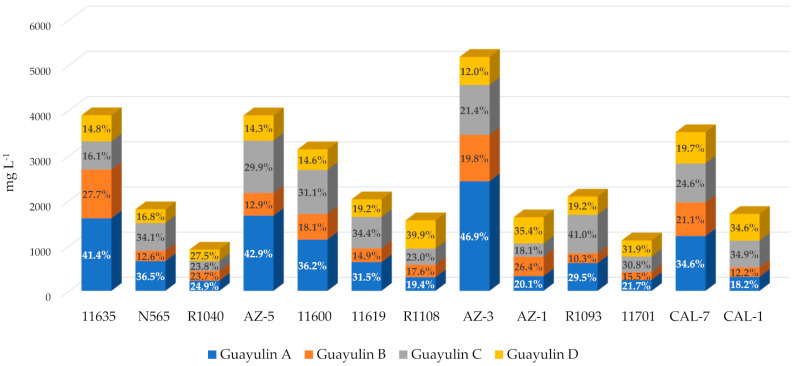
Guayulin content (expressed as mg L^−1^ of guayulin A) in leaves at November harvest for each accession.

Several things became evident when analyzing leaf samples in November. Guayulin content in the leaves in November was, surprisingly, not low (between 1000 and 4000 mg L^−1^ for all accessions except AZ-3, Figure 6) compared with the stem values (between 2000 and 4000 mg L^−1^ for all accessions except for R1040 and CAL-7, Figure 5b).

Guayule leaves have been always considered a by-product and, on their own, never a possible source of valuable compounds [30,31]. The proportion between guayulins was more balanced than in the case of the stems. Indeed, all the guayulins were present in all the accessions and their distribution was much more stable (Figure 6) as compared with the marked prominence of guayulin A in the September stems (Figure 5a) and guayulin C in the November stems (Figure 5b). This more balanced distribution was more evident when the content of each guayulin was presented in relation to the content of one of them, for example, guayulin A (Table 3).

The median of the values with respect to guayulin A was 0.61 for guayulins B and D, and 0.93 for guayulin C (Table 3). Less heterogeneity in behavior was appreciated in the stem analysis, but much greater dispersion. In the leaves, guayulin B was always the least represented and guayulin C had the highest values (Table 3). Guayulin B was much lower in concentration than guayulin A (ratio = 0.14, Table 3), guayulin D was higher in proportion to guayulin A (ratio = 1.43), and the higher ratio was for guayulin C (6.70).

## 3. Discussion

It is well known that the content of guayulin changes significantly throughout the year depending on the phenological phase of the plant [17], the part of the plant [3] and the variety [32] analyzed, and whether it is a pure guayule line (*Parthenium argentatum*) or a hybrid (with *P. tomentosum* or *P. fruticosum*) [33]. Regarding differences due to hybridization, a similar behavior was also found regarding some hybrids (CAL-1 and AZ-3) (Figure 4), which is different from the other hybrids and for pure guayule varieties (CAL-7 and R1040) that are phylogenetically related [16].

The presence of four guayulins (A, B, C, and D) on stems from 13 different guayule accessions or their hybrids was characterized, and a concentration of total guayulins was found in the range of 3542–13,311 mg L^−1^ at the end of summer, expressed as guayulin A content. During this study, the difficulty emerged when no commercial standards of guayulins were available. Those that had been used by other authors before were no longer available [1]. It was only possible to isolate guayulin A in our laboratory with enough purity to be considered as standard. Guayulin B, C and D content were expressed as guayulin A concentration.

**Figure 7:**
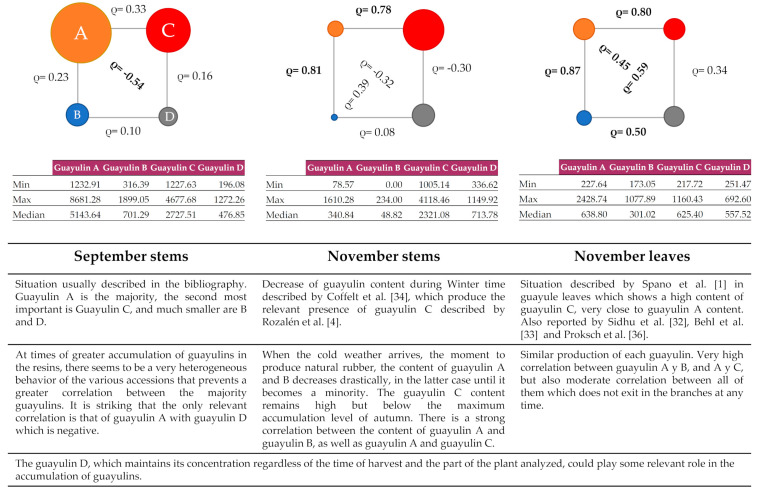
Summary diagram of the content of each guayulin in the various situations. Pearson’s correlation coefficient (ρ) is represented among the different circles that represent each guayulin. In the case of stems, all the circles are significantly different at a 95% confidence level when guayulin contents between September and November are compared by analysis of variance.

For guayulin A, the values reported here between 1233 and 8681 mg L^−1^ are easier to compare with other studies as there are many more examples in the literature, but not for the sum of the four. These values are comparable with those determined by other authors (27–3629 mg L^−1^ [17]; 514–5076 mg L^−1^ [32]; 3410–6553 mg L^−1^ [3]; and 7800–9830 mg L^−1^ [1]. A large decrease in total guayulin content was observed in the following sampling conducted in November (2018), which was more prevalent for guayulins A and B (Figure 7).

This reduction may be attributed to the drop in temperature. Comparing pre-harvest temperature between September and November sampling, a sustained minimum temperature difference of at least 6 °C was observed for much of the pre-harvest period, increasing to over 10 °C in the last ten days. This decrease in guayulin content has been previously described. Coffelt et al. [34] found a common behavior for two varieties, 11591 and AZ-2, in the 2003–2004 winter season. They found that in November 2003, there was a slowdown in the rapid increase in guayulins A and B that had taken place to July of that year, decreasing until the new sampling of March 2004 and increasing again remarkably in July 2004.

Thus far, no pattern had been found that could explain the changes detected throughout the year. Based on these results, it can be stated that the arrival of winter is accompanied by a decrease in guayulin content. Guayulin A is considered a reservoir of cinnamic acid that is subject to metabolic reversion when it is needed in the plant [17] to synthesize the many secondary metabolites that are produced from this compound [35]. As the major guayulin compound, its loss is more evident, but guayulin B also decreases proportionally in the month of November although its moiety is different, *p*-anisic acid. This suggests that the guayulins do not disappear because they are a storage form of the cinnamic and anisic acids, as could be thought at first, but because the plant needs the terpenoid center.

The inclusion of very different accessions in this study, including hybrids, likely explains why it is challenging to find correlations between the values for guayulin A and values of other guayulins in the September sample (Figure 7). Their particular accumulation behavior makes it difficult to find such correlations. On the other hand, in November, which is a dynamic situation as guayulins are produced but also disappear, it is possible to find strong correlations between guayulin A and guayulin B (ρ = 0.81) or C (ρ = 0.79) (Figure 7). However, when correlations are established for the calculated ratios November/September for each guayulin, strong correlations are found between guayulins A and B (0.97), C and D (0.76), B and C (0.68) and A and C (0.59) (Table 2). This indicates that the evolution of guayulins is linked. Guayulins A and B decrease in all accessions from September to November, while guayulin C increases in R1040 and guayulin D changes heterogeneously attending to the accession, increasing in AZ-3, AZ-1, 11635, 11701, 11600, N565 and R1108.

Differences in the proportion of guayulins also depend on the plant tissue analyzed [3]. In our study, plant processing was carried out in such a way that the plants were dried whole and only later were the leaves separated from the stems. It was our intention that all material was treated in the same manner such that the levels of guayulins would not be treatment-dependent. A surprising result when analyzing the leaves was that the guayulin content was quite similar, with a remarkable correlation even in the relation between guayulin A and D or guayulin B and C (Figure 7), which are structurally distinct from one and other (Figure 1). The finding of similar concentrations of guayulins in the leaves has previously been described by other authors. Proksch et al., [36] found that whereas guayulin A was 3–5 times higher than guayulin B in the stem the ratio was essentially 1:1 in the leaves. Additionally, Sidhu et al. [32] found a similar behavior when studying different parts of the GILA guayule cultivar in samples collected between December and January, with the leaves showing the closest relationship between the concentration of guayulin A and B. The higher production of guayulin A and B in the leaves than in the stems in November may suggest that the leaves production of these compounds is not able to replace their disappearance in the accumulation tissue the stems. 

It has always been believed that the guayule leaf is essentially a by-product of the extraction of rubber from this plant because no use has been found for it beyond an organic soil amendment [20]. Recently several authors [37,38] have postulated that it might be valorized by using it to extract phenolic compounds. Another possible use would be the extraction of guayulins, as their content is not as low as one might expect. Indeed, values of 228–2429 mg L^−1^ have been found, which is similar to the mg L^−1^ determined by other authors [36]. This might open a new line of exploitation for guayule that would facilitate its commercialization. A caveat to this is that it clearly depends on whether it is easier or more difficult to extract these compounds, which is presumed to have fungicidal and acaricidal activity [7] from the leaves or stems. *A priori* the fact that the leaves have less rubber content could facilitate the extraction of the guayulins.

Guayulin D, which has received little attention thus far and which maintains its concentration regardless of the time of harvest and the part of the plant analyzed (Figure 7), could play some relevant role in the accumulation of guayulins. 

## 4. Conclusions

The drop in autumn–winter temperatures that is known to trigger natural rubber synthesis in guayule stems also causes a marked decrease in the contents of guayulin A and B. It has been shown that Guayulin A, which has been the major guayulin until now, is no longer the most abundant, but maintains a close relationship with guayulins B and C in the stems. At the same time, the production of the four guayulins in the leaves is very similar and the concentration of guayulins A and B is higher than in the stems. Therefore, if guayulins are translocated from the leaves to the stems, they are being used at a greater speed than they are produced and translocated, as they are below the previous level of accumulation (September) and also below the level synthesized in the leaves. Guayulin D could play a relevant role in the accumulation of the different members of the family. Indeed, it is the only compound that maintains its concentration irrespective of the time of harvest or the part of the plant analyzed and is related to the content of guayulin A during the active period of synthesis and storage of the sesquiterpenes. Although it cannot be sure that the loss of the guayulins with the arrival of the winter means that they are involved in the synthesis of natural rubber, it opens a new avenue to explore their potential contribution in the future.

## 5. Materials and Methods

### 5.1. Guayule

A set of 13 guayule and hybrid accessions (11600, 11619, 11635, 11701, AZ-1, AZ-3, AZ-5, CAL-1, CAL-7, N565, R1040, R1093 and R1108) were selected for the present study. Cultivars were 18 months old and were grown in Santa Cruz de la Zarza (Toledo, Spain) in three replicate plots. Four homogeneous and adjacent plants for each of replicate were randomly selected and harvested in September (SH) and November of 2018 (NH). Plants were manually harvested by cutting them 5 cm above the ground and they were packaged in kraft bags. Samples were dried in the laboratory for 48 h at 60 °C to achieve 12% moisture content. The moisture content of the samples were determined with a halogen lamp moisture balance model XM-120T (Cobos, Barcelona, Spain) at 105 °C, and when moisture loss was less than 0.1% in 180 s, it was considered that samples had reached constant mass. Then, leaves and flowers were manually removed and the dry biomass weight was calculated from the dried branches and leaves. Branches were cut into pellets of about 1 cm in length with a manual cutter and were grounded in a 2-step procedure: first, 2-mm-sized particles were obtained using a hammer grinder and then 0.5-mm-sized particles were obtained with a centrifuge grinder. Leaves were ground directly to 0.5 mm in one step. Dried ground samples were stored in screw cap closed glass vessels at room temperature.

### 5.2. Resin Extraction

The extraction of resin was performed in a BUCHI E-914 Speed Extractor (Barcelona, Spain). A sample of 1.5 ± 0.005 g of guayule (0.51-mm-sized particles of guayule branches or guayule leaves) was weighed and homogenized with approximately 32 g of sand as a dispersing agent and packed with 37 g of sand above and below to fill the stainless steel 80-cm^−3^ extraction cell, leaving a 1-cm free space. A cellulose acetate filter was also placed at the top and bottom of each cell to avoid sample particles leaking into the cells. The resin extraction conditions using acetone as a solvent were as follows: temperature 40 °C, pressure 100 bar, hold 3 cycles 10/20/30 min, 1-min heat-up, 3-min discharge, 2-min flush with solvent, and 5-min flush with N_2_. 

Resin was collected in a 240-cm^−3^ flask and transferred to a pre-weighed flask and equilibrated for 30 min in a desiccator. Solvent evaporation was carried out in a parallel Multivapor BUCHI P-6 system (Postfach, Switzerland) at 50 °C and 150 mbar. After evaporation, the pre-weighed flasks were maintained for 60 min in a desiccator before the final weighing. The resin percentage was then determined gravimetrically considering the dry weight. Each sample was extracted twice. 

### 5.3. Guayulin Quantification by High-Performance Liquid Chromatography–Diode Array Detection

Twenty microliters of resin dissolved in ethanol (10 mg mL^−1^) and filtered (0.22 µm) was injected into an Agilent 1200 high-performance liquid chromatography (HPLC) system (Agilent Technologies, Palo Alto, CA, USA) equipped with a diode array detector (DAD) (Agilent Technologies, G1315D). Separation was performed at 30 °C on a reverse-phase ACE Excel 3 C18-PentaFluorPhenyl (PFP) column (150 × 4.6 mm, 3 μm particle size) protected with an ACE Excel HPLC Pre-column Filter (0.5 μm particle size) (both from Advanced Chromatography Technologies Ltd., Reading, Berkshire, UK). The solvents were Milli Q-grade water (solvent A) and acetonitrile (solvent B). The elution gradient for solvent B was as follows: 0 min, 60%; 10 min, 60%; 20 min, 80% and hold 5 min; 35 min, 100% and hold 2 min. Agilent ChemStation software (version B.03.01) was used for the quantification of the four guayulins (A, B, C and D) using a guayulin A standard five level calibration curve (0.1–250 mg L^−1^, r^2^ = 0.997) which its limit of detection and quantitation was 32.37 µg L^−1^ (LOD) and 116.01 µg L^−1^ (LOQ).

The guayulin A standard (98% purity) was isolated in our laboratory due to the absence of commercial standards. Low molecular-weight guayule rubber (LMWGR) was removed from the resin extract [39]. Then, free-rubber resin was fractionated using flash chromatography (VersaFlash Station System I equipped with a Versaflash cartridge, 23 mm × 110 mm, containing Spherical C18 bonded silica 30 g, 20–45 μm) with acetonitrile: water (80:20) as solvent and a flow rate of 20 cm^−3^/min. The fractionation procedure was followed with HPLC-DAD. The guayulin A fraction was evaporated under vacuum and crystalized from hexane/chloroform, as described [23]. Molecular weight and mass fragmentation patterns were confirmed by liquid chromatography–mass spectrometry. Guayulin B, C and D were tentatively identified by their characteristic UV absorption spectra (maximum at 256 nm for guayulin B and D; maximum at 276–278 nm for guayulin C) and retention time [4].

### 5.4. Data Analysis

Statistical analysis was performed with IBM SPSS Statistics v25 [40]. Multivariate analysis was performed to compare resin content (% R) and total guayulins per plant (in mg L^−1^) of the 13 accessions attending to the harvest time (September and November).

We also examined the relationship between the potential drop in guayulin content and the genetic origin of the 13 accessions using the average values introduction method, generating neighbor-joining trees. The genetic characteristics considered were according to: (1) hybridization (11 guayule and 2 non-mariola hybrids (NMH)); (2) ploidy attending to Ilut et al. [16], considering all the potential types; (3) ancient groups established following [28] (1942–1960, 1961–1982 and 1983–2008), and (4) November/September ratio calculated for resins and total guayulins.

The November/September ratio for the different guayulins (A, B, C and D) at every harvest date and the relationship of every guayulin with guayulin A were also determined and represented as boxplots. Pearson’s correlation analysis was performed for the November/September ratios between the different combinations of guayulins in order to compare their behavior from the end of summer to the fall. Finally, multivariate analysis was performed to compare guayulin content in both stems and leaves at every harvest.

## Figures and Tables

**Table 1:** Relationship between guayulins (A, B, C and D) for each harvesting time depending on guayule accession.

Accession	September Sampling				November Sampling			
	A/B	C/D	A/C	B/D	A/B	C/D	A/C	B/D
CAL-7	1.76	1.54	0.63	0.55	1.76	1.54	0.63	0.55
AZ-3	2.27	2.23	3.06	3.01	2.27	2.23	3.06	3.01
CAL-1	3.08	2.66	1.20	1.04	3.08	2.66	1.20	1.04
11701	3.27	3.09	2.28	2.15	3.27	3.09	2.28	2.15
R1093	5.04	4.63	1.71	1.57	5.04	4.63	1.71	1.57
11635	6.34	5.89	1.86	1.73	6.34	5.89	1.86	1.73
R1040	6.57	6.09	1.84	1.71	6.57	6.09	1.84	1.71
AZ-5	6.75	6.98	1.15	1.19	6.75	6.98	1.15	1.19
AZ-1	6.92	5.53	0.47	0.37	6.92	5.53	0.47	0.37
11619	8.11	7.67	1.46	1.38	8.11	7.67	1.46	1.38
11600	8.97	8.65	2.75	2.65	8.97	8.65	2.75	2.65
R1108	12.90	12.07	1.60	1.50	12.90	12.07	1.60	1.50
N565	13.76	11.82	3.74	3.22	13.76	11.82	3.74	3.22
Min	1.76	1.54	0.47	0.37	1.63	1.02	0.08	0.03
Max	13.76	12.07	3.74	3.22	15.41	6.99	0.39	0.30

**Table 2:** Comparison of guayulin content in the September and November harvest for each accession.

Accessions	A_N_/A_S_	B_N_/B_S_	C_N_/C_S_	D_N_/D_S_
CAL-7	0.06	0.04	0.51	0.75
AZ-3	0.03	0.04	0.83	1.82
CAL-1	0.13	0.13	1.31	1.87
11701	0.19	0.17	1.24	1.68
R1093	0.21	0.21	0.93	1.10
11635	0.05	0.04	0.61	1.07
R1040	0.25	0.24	1.17	1.36
AZ-5	0.04	0.00	0.50	1.07
AZ-1	0.14	0.10	0.61	1.06
11619	0.13	0.10	0.70	1.10
11600	0.05	0.04	0.86	1.68
R1108	0.06	0.00	0.67	1.27
N565	0.09	0.08	1.08	1.83
Median	0.09	0.08	0.83	1.27
Min	0.03	0.00	0.50	0.75
Max	0.25	0.24	1.31	1.87

**Table 3:** Ratio of guayulin B, C and D versus guayulin A content in leaves and stems.

Accession	Leaves			Stems		
	B/A	C/A	D/A	B/A	C/A	D/A
11600	0.50	0.86	0.40	0.09	6.34	1.43
11619	0.47	1.09	0.61	0.09	3.63	0.74
11635	0.67	0.39	0.36	0.14	7.10	2.13
11701	0.71	1.42	1.47	0.27	2.80	1.23
AZ-1	1.31	0.90	1.77	0.11	9.30	2.95
AZ-3	0.42	0.46	0.25	0.61	10.74	10.56
AZ-5	0.30	0.70	0.33	0.00	10.85	3.34
CAL-1	0.67	1.91	1.90	0.32	8.57	4.60
CAL-7	0.61	0.71	0.57	0.35	12.79	12.22
N565	0.35	0.93	0.46	0.06	3.34	0.48
R1040	0.95	0.96	1.10	0.15	2.56	0.49
R1093	0.35	1.39	0.65	0.20	2.60	0.67
R1108	0.91	1.18	2.05	0.00	6.70	1.05
Median	0.61	0.93	0.61	0.14	6.70	1.43
Min	0.30	0.39	0.25	0.00	2.56	0.48
Max	1.31	1.91	2.05	0.61	12.79	12.22

## Data Availability

The data presented in this study are available on request from the corresponding author.
